# Subject-Controlled, On-demand, Dorsal Genital Nerve Stimulation to Treat Urgency Urinary Incontinence; a Pilot

**DOI:** 10.3389/fnins.2016.00024

**Published:** 2016-02-09

**Authors:** Hendrikje M. K. van Breda, Fawzy F. Farag, Frank M. J. Martens, John P. F. A. Heesakkers, Nico J. M. Rijkhoff

**Affiliations:** ^1^Department of Urology, Radboud University Nijmegen Medical CentreNijmegen, Netherlands; ^2^Department of Urology, Sohag University HospitalSohag, Egypt; ^3^Center for Sensory-Motor Interaction, Aalborg UniversityAalborg, Denmark

**Keywords:** dorsal genital nerve, electrical stimulation, urinary incontinence, urgency, overactive bladder syndrome

## Abstract

**Objectives:** To evaluate the effect of subject-controlled, on-demand, dorsal genital nerve (DGN) stimulation on non-neurogenic urgency urinary incontinence (UUI) in a domestic setting. **Materials and Methods:**Non-neurogenic patients >18 years with overactive bladder symptoms and UUI were included. Exclusion criteria were mainly stress urinary incontinence. Patients underwent 1 week of subject-controlled, on-demand, DGN stimulation, delivered by a percutaneously placed electrode near the DGN connected to an external stimulator (pulse-rate 20 Hz, pulse-width 300 μs). Patients activated the stimulator when feeling the urge to void and stimulated for 30 s. The amplitude was set at the highest tolerable level. A bladder diary including a severity score of the UUI episodes/void (scores: 0 = none, 1 = drops, 2 = dashes, 3 = soaks) and a padtest was kept 3 days prior to, during, and 3 days after the test period. The subjective improvement was also scored.

**Results:** Seven patients (4 males/3 females) were enrolled, the mean age was 55 years (range 23–73). Six completed the test week. In the remaining patient the electrode migrated and was removed. 5/6 finalized the complete bladder diary, 1/6 recorded only the heavy incontinence episodes (score = 3). 4/6 completed the padtest. In all patients who finalized the bladder diary the number of UUI episodes decreased, in 3/5 with ≥60%. The heavy incontinence episodes (score = 3) were resolved in 2/6 patients, and improved ≥80% in the other 4. The severity score of the UUI episodes/void was improved with ≥ 60% in 3/5 patients. The mean subjective improvement was 73%.

**Conclusion:** This feasibility study indicates that subject-controlled, on-demand DGN stimulation using a percutaneously placed electrode is possible over a longer time period, in a home setting, with a positive effect on non-neurogenic overactive bladder symptoms with UUI. Although the placement is an easy procedure, it is difficult to fixate the electrode to keep it in the correct position. Improvements in hardware, like a better fixated electrode and an easy to control stimulator, are necessary to make SODGNS a treatment possibility in the future.

## Introduction

In patients with urgency urinary incontinence (UUI) the time between the sensation to void and reaching the toilet is mostly too short to prevent incontinence. UUI is often challenging to treat and affects the quality of life of many people negatively.

Conservative treatment comprises bladder and behavioral training with or without anticholinergics or mirabegron. Side effects, like dry mouth and constipation, strongly decrease compliance of patients to treatment, are an important reason to discontinue treatment (Veenboer and Bosch, 2014). More invasive treatments can be considered in patients who do not tolerate side effects or who do not respond satisfactorily. Intravesical injections of Botulinum toxin and neuromodulation are examples of more invasive or surgical treatment. Drawbacks of intravesical injections of Botulinum toxin are the temporary effect which demands repeated injections and the risk for residual urine or urinary retention with the need for self-catheterisation. With regard to neuromodulation are Percutaneous Tibial Nerve Stimulation (PTNS) and Sacral Nerve Stimulation (SNS) nowadays widely adopted as a treatment which diminishes UUI (van Kerrebroeck et al., 2007; Peters et al., 2010, 2013; Groen et al., 2011). With PTNS a moderate or markedly result is seen in 55% of the patients (Peters et al., 2010), however PTNS is a rather time consuming therapy. Patients need to visit the office once a week for 12 weeks. A electrode needle has to be inserted correctly for each stimulation session. To maintain the treatment effect patients need to continue their visits more than once a month (Peters et al., 2013). With SNS therapy the success rate, ≥50% improvement, is around 62–68% (van Kerrebroeck et al., 2007; Groen et al., 2011). Complete continence is reached in only 15% of the patients (Groen et al., 2011).

Dorsal genital nerve (DGN) stimulation has been used in research settings and seems to be promising for future use in clinical care (Goldman et al., 2008; Martens et al., 2011a; Farag et al., 2012). The DGN, being the dorsal penile nerve (DPN) or clitoral nerve (DCN), is a terminal branch of the pudendal nerve. Previous studies have shown that DGN stimulation can inhibit involuntary bladder contraction in an acute investigational setting (Lee et al., 2005; Martens et al., 2011b). When inhibition of the detrusor contraction can be effectuated with on demand DGN stimulation in a domestic setting, complete continence could theoretically be achieved. Using on demand stimulation to inhibit an involuntary detrusor contraction the patient gains time to reach the toilet. A study on subject-controlled, on-demand, dorsal genital nerve stimulation (SODGNS) through self-adhesive surface electrodes in neurogenic patients with detrusor overactivity who had intact bladder sensations showed a reduction in their UUI episodes during 5 days of stimulation at home (Opisso et al., 2013). Another study used a percutaneous electrode for continuous stimulation at home in non-neurogenic women with UUI, who also reported a reduction in UUI episodes (Goldman et al., 2008). The results of subject-controlled on-demand stimulation with an implanted electrode in non-neurogenic patients with UUI in daily life are not known yet. Previous studies used continuous stimulation instead of on-demand stimulation, included patients with neurogenic detrusor overactivity instead of non-neurogenic UUI, or only acute effects of on-demand stimulation in a laboratory setting were used instead of application in a out of hospital setting with daily activities (Martens et al., 2011b; Opisso et al., 2013).

The purpose of the current study was to investigate the effect of SODGNS on overactive bladder (OAB) with UUI in a home testing period using an implanted electrode in non-neurogenic patients with refractory OAB and UUI.

## Materials and methods

The study was approved by the accredited medical research ethics committee, Committee on Research Involving Human Subjects region Arnhem-Nijmegen (The Netherlands) and conducted according to the principles expressed in the declaration of Helsinki. Written informed consent was obtained. The inclusion criteria were: age >18 years old, OAB with UUI, and more than 4 UUI episodes a day, patients who are willing and aiming to follow all requirements of the protocol. The exclusion criteria: Neurogenic OAB, pure stress urinary incontinence, neurological disease, skin lesions at the implantation site, an increased risk of infections, poor wound healing, bleeding tendency, DGN or pudendal nerve or sacral root lesions, a cardiac pacemaker, peripheral neuropathy, and pregnancy.

**Figure 1:**
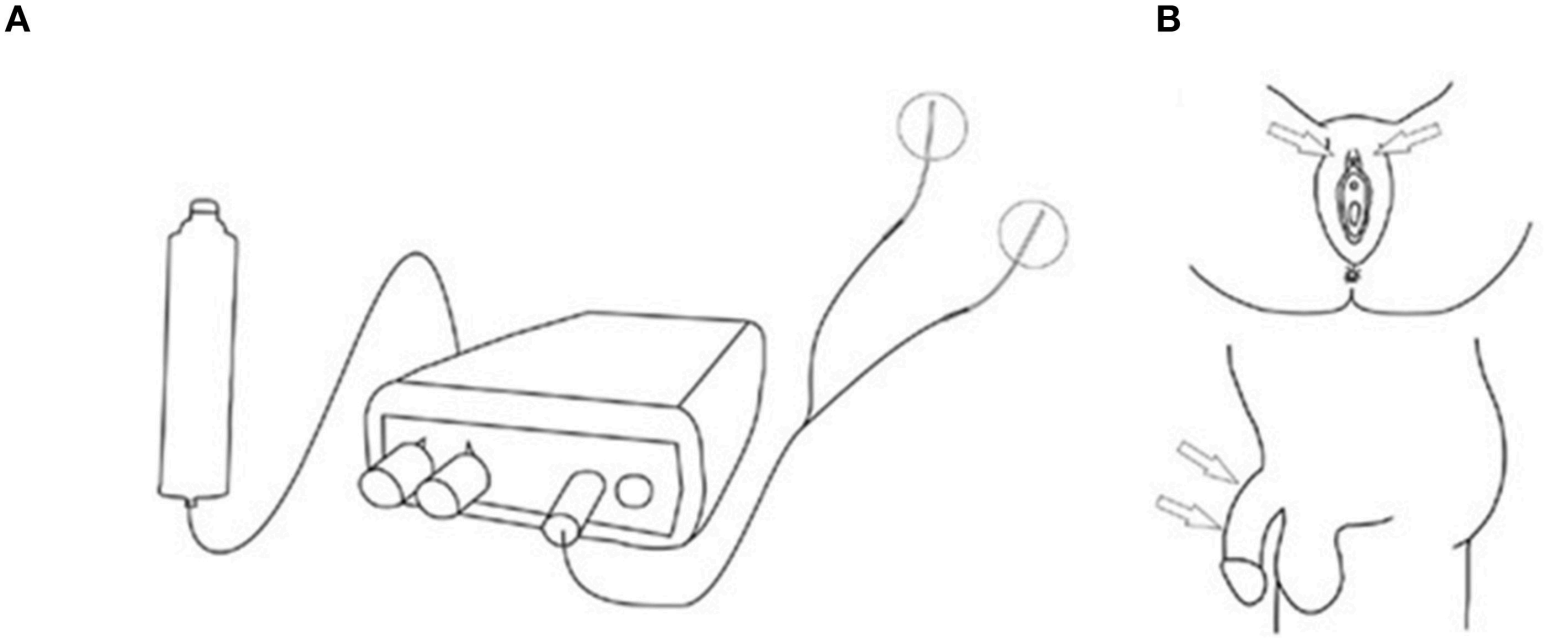
**Stimulation setup self-adhesive skin electrodes. (A)** Stimulation setup. **(B)** Stimulation sites. From Opisso et al. (2013), with permissions.

Patients on anticholinergics had a wash-out period of 2 weeks. All patients kept a bladder diary, a pad test and scored once daily the Patient Perception of Intensity of Urgency Scale (PPIUS), 3 days prior to and during the 7 days home stimulation days. The bladder diary contained frequency, micturition volume, intake, and the severity of UUI estimated by the Severity Score of Incontinence Episodes (SSIE) (0 = none, 1 = drops, 2 = dashes, 3 = soaks). At the end of the SODGNS week, the subjective improvement was scored. All subjects underwent a test period of SODGNS for 4 h using self-adhesive skin electrodes. In males we used 25 mm-diameter round PALS electrodes, (Axelgaard manufacturing Co., Fallbrook, CA,). The cathode and anode were placed, proximally and distally, respectively, on the dorsum of the penile shaft. In females a PALS electrode was positioned next to the clitoris as anode and a Neuroline700 (Ambu, A/S, Ballerup, Denmark) as cathode on the clitoris (Figure 1).

**Figure 2:**
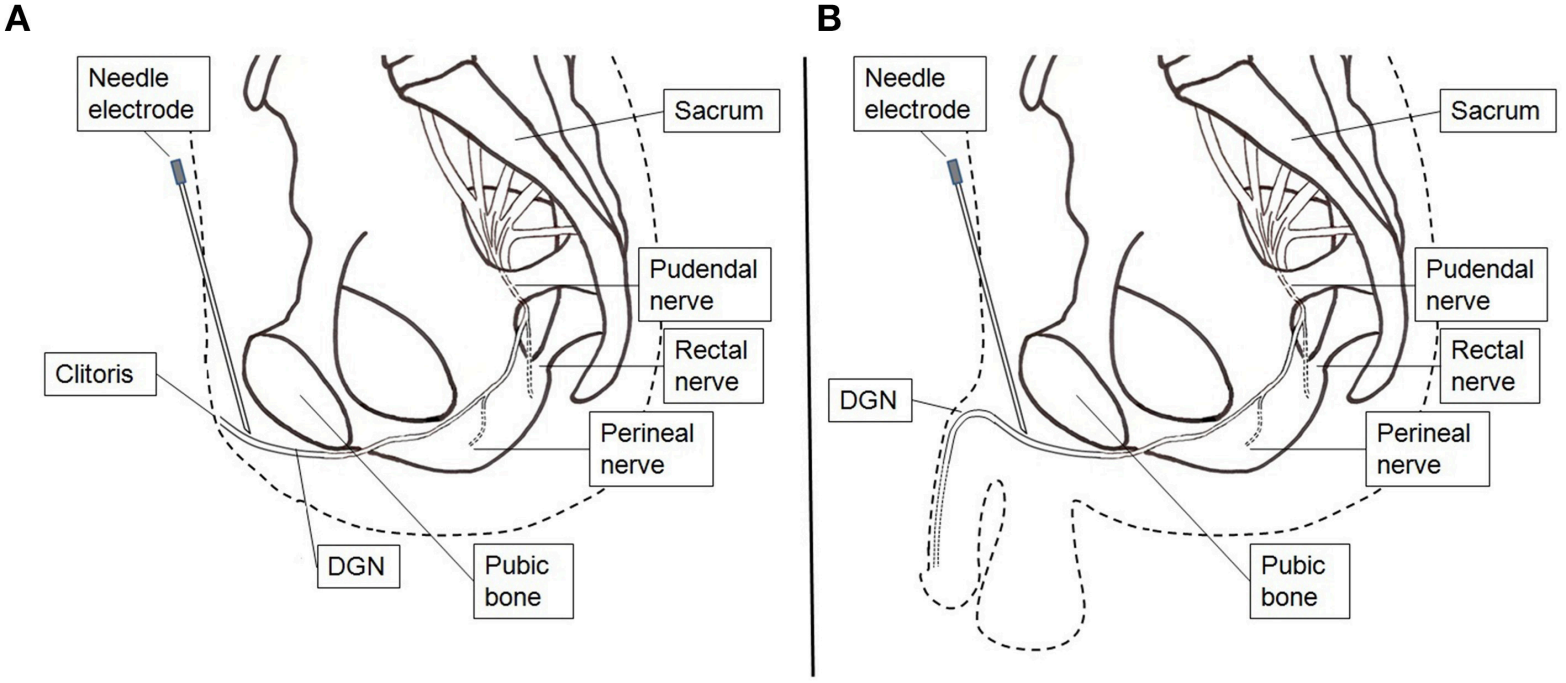
**Stimulation setup percutaneous lead electrode insertion**. **(A)** Female. **(B)** Male. A hollow needle electrode is used to find the correct position and insert the lead electrode.

The electrodes were connected to a handheld battery powered current controlled stimulator (Odstock O2CHS, Salisbury, Wiltshire, UK). Stimulation was initiated by pushing a button and lasted for 30 s. They started stimulation every time as soon as they felt the need to go to the toilet to pass urine. However, when they were at the toilet and wanted to void, they did not activate the stimulator and voiding could proceed as normal. The stimulator was set to square pulses at a pulse-rate of 20 Hz and a pulse-width of 300 μs. The stimulation amplitude was set at the highest tolerable level. If during the test period with surface electrode stimulation the UUI decreased subjectively they underwent percutaneous placement of an electrode (Medtronic InterStim Model 3057 Test Stimulation Lead, Minneapolis, USA) under local anesthesia (lidocaine 1%). The electrode placement was performed in the outpatient clinic. Firstly, a needle electrode was inserted in the pubic area in the direction of the clitoris or the penile base. Test stimulation was applied and patients were asked where the stimulation was felt. If the patient reported a sensation localized at the clitoris or at the glans penis, it was concluded that proper placement was obtained (Figure 2). After proper needle positioning the lead was introduced, fixated to the skin, and connected to an external voltage controlled stimulator (Medtronic inc., Model 3625, Minneapolis, USA). The stimulator was set to square pulses at a pulse-rate of 20 Hz and a pulse-width of 300 μs. This stimulator was activated by turning the amplitude control button to its maximum and was turned off by turning the control button back to 0. The maximum was limited to the patient's highest tolerable level. The patients were sent home for 1 week. Conform the instructions for the test period patients were asked to stimulate every time as soon as they felt the need to go to the toilet to pass urine for 30 s. Improvement was defined as ≥60% reduction in UUI episodes.

## Results

Seven patients, 4 males and 3 females were enrolled. The mean age was 55 years old (range 24–73). All patients had a positive result on their UUI during the test-stimulation with skin electrodes and subsequently an electrode was implanted. The implantation took 10–40 min. In four patients the electrode dislocated after stepping off the treatment bench where after upon stimulation the sensation in the desired location was gone. In those patients a new electrode was placed.

At the time of lead placement, the stimulus amplitude at which the subject first felt the stimulus (sensation threshold) was 1.6 ± 0.8 V (range 1–3 V), and the maximum tolerable amplitude was 7.6 ± 2.2 V (range 6–10 V). Six out of seven patients completed the protocol. One patient did not feel the sensation in the proper position after 2 days of home stimulation, likely due to migration of the electrode, and the electrode was removed. At the end of the week of stimulation the sensation threshold amplitude was higher but not clinical significantly (1.8 ± 0.4 V, range 1–2 V).

Three out of six patients reported that the location of sensation had slightly changed during the week of home stimulation, where after the subjective effect of stimulation on their UUI was slightly less.

Of the six patients who completed the week of home stimulation, 4/6 completed the padtest, all completed the PPIUS, 5/6 finalized the complete bladder diary and the remaining one recorded only the heavy IE (3 = soaks) and the nighttime frequency in her bladder dairy.

Results are shown in Table 1, and the results per patient per day are shown in Table 2.

**Table 1:** **Mean of variables of the days prior to stimulation and the days during of SODGNS**.

**Patient**		**1**	**2**	**3**	**4**	**5**	**6**
**Gender**		**M**	**M**	**F**	**F**	**M**	**F**
Mean number of IE/day	Before SODGNS	14.0	6.0	–	8.3	17.7	8.0
	SODGNS	13.5	0.7	–	3.3	14.8	0.5
	Improvement (%)	4	88	–	60	16	94
Mean heavy leaks/day	Before SODGNS	1.0	1.0	3.3	2.7	5.0	2.3
	SODGNS	0.2	0.0	0.3	0.5	1.0	0.0
	Improvement (%)	80	100	91	81	80	100
Mean SSIE/void	Before SODGNS	2.0	0.6	–	1.9	2.2	2.2
	SODGNS	1.6	0.1	–	0.5	1.3	0.1
	Improvement (%)	20	83	–	74	41	95
Mean padtest (g/day)	Before SODGNS	63.0	–	402.7	76.0	–	313
	SODGNS	16.7	–	295.2	55.8	–	3.3
	Improvement (%)	73	–	27	27	–	99
Mean daytime frequency	Before SODGNS	14.0	10.7	–	8.3	15.7	7.0
	SODGNS	14.0	8.0	–	8.0	13.8	5.0
	Improvement (%)	0	25	–	4	12	29
Mean nighttime frequency	Before SODGNS	0.3	3.3	2.7	1.0	2.0	1.0
	SODGNS	0.0	2.2	1.5	1.0	1.3	0.2
	Improvement (%)	100	33	44	0	35	80
Mean PPIUS	Before SODGNS	3.0	3.5	4.0	3.8	3.2	4.0
	SODGNS	2.0	1.3	2.5	2.1	3.3	2.5
	Improvement (%)	33	67	38	45	−3	38
Subjective improvement (%)		90	70	70	80	30	100

*SODGNS, subject-controlled, on demand dorsal genital nerve stimulation; SSIE, severity score of incontinence episodes; PPIUS, patient perception of intensity of urgency scale*.

**Table 2:** **Mean SSIE/void per day and mean Pad test per day**.

**Patient**			**1**	**2**	**3**	**4**	**5**	**6**
**Gender**			**M**	**M**	**F**	**F**	**M**	**F**
SSIE/void	Before SODGNS	1	2.1	0.9	–	1.4	2.2	2.3
		2	1.9	0.5	–	2.1	2.1	2.4
		3	2.1	0.4	–	2.1	2.3	2.0
	SODGNS day	1	1.3	0.0	–	1.0	1.3	0.0
		2	1.7	0.0	–	0.3	1.2	0.0
		3	1.6	0.1	–	0.6	1.5	0.0
		4	1.7	0.2	–	0.4	1.3	0.2
		5	1.6	0.1	–	0.3	1.2	0.3
		6	1.7	0.1	–	0.6	1.2	0.2
Pad test (g/day)	Before SODGNS	1	59	–	450	114	–	315
		2	59	–	450	38	–	315
		3	57	–	338	–	–	324
	SODGNS	1	13	–	–	160	–	0
		2	32	–	336	6	–	0
		3	15	–	364	64	–	0
		4	15	–	200	70	–	8
		5	11	–	334	12	–	5
		6	14	–	242	23	–	7

*SODGNS, subject-controlled, on demand dorsal genital nerve stimulation; SSIE, severity score of incontinence episodes*.

The heavy IE (3 = soaks) resolved completely in 2/6 patients, and 4/6 patients had a ≥80% improvement of the heavy leaks (91, 81, 80, and 80%).

In all of the five patients who finalized the complete bladder diary the number of UUI episodes decreased. In 3/5 it improved with ≥60% (94, 88, 60%) the other two improved with 16 and 4%. The SSIE/void was improved with at least 60% in 3/5 patients (95, 83, 74%) the other 2/5 patients had an improvement of 41 and 20%.

In 4/5 patients the daytime frequency decreased. The mean number of voids per day with and without SODGNS were 10 (range 5–14) and 11 (range 7–16), respectively. The nighttime frequency decreased in 5/6 patients, and was unchanged in the remaining one.

The voided volume per day during the test week was comparable to the days before the test week, 1809 (±346) ml/day respectively 1821 (±553) ml/day. The mean voided volume (MVV) increased in 4/5 patients. The MVV with SODGNS was 223 (±148) ml and without SODGNS 184 (±93) ml.

The PPIUS improved in 5/6 patients. All patients used pads for their incontinence, four completed the padtest. Of the four subjects who completed the padtest, 2 had at least 60% improvement (99 and 73%) the other 2 had both 27% improvement. The subjective improvement reported by the patients were: 100, 90, 80, 70, 70, and 30%.

Patients mentioned that the SODGNS had a better effect, when the stimulator was activated without delay in response to the urge to void. They also mentioned that with the button they had to turn activating the stimulator which was used during the test week, was more inconvenient compared to the pushbutton, which was used during the 4 h test period with the skin electrodes.

After the stimulation week, one patient reported that the effect on the UUI was immediately gone and 5/6 patients reported that the effect waned over time. They reported to have a decreasing effect over a time period from 1–7 days.

During the week of home stimulation 1 patient experienced a mild muscle pain in her pelvic floor. One patient experienced a headache at the first day and one patient had 1 day of chest pain, cardiac evaluation showed no abnormalities. At explantation of the electrode 4/6 patients had a temporary small hematoma and in one patient some redness at the implantation site was observed. All resolved spontaneously.

## Discussion

This pilot study demonstrates a decrease in UUI with subject controlled, on demand DGN stimulation using a percutaneously placed electrode in patients with idiopathic refractory UUI in a home setting. One patient was almost 100% dry, she described that she was completely dry except for the moments when she wasn't able to activate the stimulator immediately when she felt the urge to void, e.g., because she was driving her car or first had to undo her coat to reach the stimulator. The other patients also mentioned that the SODGNS had a better effect, when the stimulator was activated without delay in response to the urge to void. This was difficult to achieve in every episode since the stimulator was in most of the time in their pocket, which always gave a little delay. Patients also mentioned that the button they had to turn on for activation of the stimulator was more inconvenient compared to the pushbutton, and therefore more time consuming and causing delay. Probably, better results would have been achieved with a quicker and easier activation of the stimulator. This implies that immediate stimulation when feeling urge is important. This is in line with Opisso's report in 2008 (Opisso et al., 2008). He described that in patients with neurogenic detrusor overactivity, the sooner the onset of DGN stimulation during a detrusor contraction, the greater the likelihood of stopping the contraction and therefore preventing incontinence. Ideally, instead of manually turning or pushing a button while feeling urge, a sensor that continuously monitors bladder activity should be used to set off the conditional stimulation. In this way a closed loop system could be formed. There is ongoing research into bladder sensors, but until now no suitable device is available for clinical application (Melgaard and Rijkhoff, 2014).

Besides the decrease in UUI, the daytime and nighttime frequency also decreased with SODGNS. The mean volume/void increased. This supports the assumption that patients can suppress the urge to void with SODGNS, and gain time before they have to go to the toilet, and gain time to reach the toilet without UUI.

Another advantage of conditional compared to continuous stimulation is that the stimulation is only felt when activating the stimulator during the sensation of urge instead of feeling the stimulation continuously. The sensation of the stimulation was experienced as less annoying by the patients than the sensation of urgency. During an urgency episode the stimulation amplitude was experienced less intense, therefore the amplitude of SODGNS can be set to a higher amplitude compared to continues stimulation. With a higher stimulation amplitude a better effect on the bladder inhibition could be achieved and therefore a higher continence rate can be expected. Another further advantage is that SODGNS reduces power consumption compared to continuous stimulation due to reduction in stimulation time.

This study is performed in non-neurogenic OAB patients. Previous DGN stimulation in a domestic setting has been described in neurogenic OAB patients with comparable results (Wheeler et al., 1994; Lee and Creasey, 2002; Opisso et al., 2013). Opisso performed SODGNS, through self-adhesive skin electrodes, in patients with intact bladder sensation and described a decrease in their UUI (Opisso et al., 2013). Lee et al. (Lee and Creasey, 2002) published a case report of SODGNS with skin electrodes in a 33 year old spinal cord injured (SCI) male. SODGNS diminished his UUI episodes. Continuous DGN stimulation is described by Wheeler et al. (1994) in this study 2 males with SCI and UI due to detrusor overactivity fulfilled 2 weeks of continues DGN stimulation through surface electrodes and became continent.

Comparing this study with SODGNS to the publication of Goldman et al. (2008) who performed continuous DGN stimulation in non-neurogenic patients a stronger effect on UUI was found with SODGNS in our patients, although both studies contained only a small number of patients and results must be interpreted carefully. With continuous DGN stimulation 79% of the patients experienced a reduction in UUI episodes of whom 47% experienced a ≥50% reduction in UUI episodes. With SODGNS all patients experienced a reduction in incontinences episodes and a ≥50% reduction was seen in 60% of the patients. The number of heavy leaks decreased with ≥50% in 85% of the patients with continuous DGN stimulation, with SODGNS this was seen in all patients. Eighty-eight percent of the patients experienced a reduction in pad weight with continues DGN stimulation. Of them 76% experienced at least 50% reduction. With SODGNS all patients who completed the padtest had a reduced pad weight and half of them (2/4) with at least 50%. Although this is a pilot study with a small number of patients SODGNS might have better results as compared with continuous DGN stimulation.

In this study we used self-adhesive skin electrodes for the test period. All patients who were included for this study responded during this test period. Lee et al. (2005) showed that there is no difference between conditional DGN stimulating with surface electrodes comparing to percutaneous placed electrodes. So if SODGNS would be possible with an definitive implant, the screening period before implanting, the PNE or 1st stage tined lead for SNS, can be done with less invasive surface electrodes.

All subjects tolerated the DGN stimulation well. Patient's commends were about the usability of the technique, especially of the activation system as mentioned above. Theoretically, damage to the nerve or infection by the percutaneous electrode could occur. Only minor adverse events were noticed during short-term follow-up. Up to now no long-term results of electrodes near the DGN are available because of only acute settings or short-term follow-up in SODGNS studies, besides studies and experiences with PTNS and SNS. The setting of the current study would intervene with sexual intercourse and a complete implant with an electrode that doesn't migrate would therefore be desirable. In previous studies DGN stimulation did not evoke sexual feelings or responses (Goldman et al., 2008; Martens et al., 2011a). As patients use the stimulation only on demand, the stimulation does not interfere with intercourse as long as the stimulation is not activated by the patient, which is less likely to be needed during sexual intercourse due to sexual stimulation of the DGN. The influence of long-term DGN stimulation on sexuality is not known. Continuous DGN stimulation might theoretically desensitize sexual responses. With SODGNS this risk of desensitizing might probably be lower, due to less and intermittent stimulation.

One patient could not complete the week of home stimulation due to dislocation of the lead. Of the six patients who completed the week of SODGNS, three reported that the sensation had slightly changed from position during the week of home stimulation where after the effect subjectively deteriorated slightly. This shows that this percutaneously placed lead is not very prone to stay in the initial location. For future treatment an electrode with a more stable position is necessary. An percutaneous lead connected to an external stimulator is not ideal nor patient friendly. Further development requires at least a fully implanted system consisting of an electrode and a pacemaker, which can be activated by a wireless activator that is easy to use.

## Conclusion

This feasibility study indicates that it is possible to administer SODGNS using a percutaneously placed electrode, over a longer time period, in a home setting, with a positive effect on OAB with UUI. The electrode can be placed in an outpatient setting by a minimal invasive pre-pubic approach and is well tolerated by the patients. Although the placement is an easy procedure it is difficult to fixate the electrode to keep it in the correct position. Improvements in hardware, like a better fixated electrode and an easy to control stimulator, are necessary to make SODGNS a treatment possibility in the future.

## Author contributions

All authors participated in the concept and design of the study, the implanting procedure was done by Hv, FM, and NR. The acquisition and analysis of the data was done by Hv. All authors reviewed the article for important intellectual content, commented on the manuscript, and gave their final approval of the final manuscript.

## Funding

This study was funded by the Danish Advanced Technology Foundation and Neurodan.

### Conflict of interest statement

Fawzy F. Farag, Frank M. J. Martens and Nico J.M. Rijkhoff declare that the research was conducted in the absence of any commercial or financial relationships that could be construed as a potential conflict of interest. Hendrikje M. K. van Breda declares that she receives a research grant from Neurodan. John P. F. A. Heesakkers is a consultant for Uroplasty and Axonics. The urology department of the Radboud University Medical Centre has received research grant support from Neurodan, Uroplasty, and Medtronic.
